# Identification and Partial Characterization of Midgut Proteases in the Lesser Mulberry Pyralid, *Glyphodes pyloalis*

**DOI:** 10.1673/031.013.8101

**Published:** 2013-08-10

**Authors:** Atiyeh Mahdavi, Mohammad Ghadamyari, Reza H. Sajedi, Mahbobeh Sharifi, Behrooz Kouchaki

**Affiliations:** 1 Department of Biology, Faculty of sciences, University of Guilan, Rasht, Iran; 2 Department of Plant Protection, Faculty of Agricultural Sciences, University of Guilan, Rasht, Iran; 3 Department of Biochemistry, Faculty of Biological Sciences, Tarbiat Modares University, Tehran, Iran

**Keywords:** chymotrypsin, digestive proteases, *Glyphodes pyloalis*, proteinase inhibitors, trypsin

## Abstract

Proteolytic activities in digestive system extracts from the larval midgut of the lesser mulberry pyralid, *Glyphodes pyloalis* Walker (Lepidoptera: Pyralidae), were analyzed using different specific peptide substrates and proteinase inhibitors. High proteolytic activities were found at pH 10.0 and a temperature of 50° C using azocasein as substrate. The trypsin was active in the pH range of 9.5– 12.0, with its maximum activity at pH 11.5. Ethylene diamine tetraacetic acid had the most inhibitory effect, and 44% inhibition was detected in the presence of this inhibitor. Phenyl methane sulfonyl floride and N-tosyl-L-phe chloromethyl ketone also showed considerable inhibition of larval azocaseinolytic activity, with 40.2 and 35.1% inhibition respectively. These data suggest that the midgut of larvae contains mainly metalloproteases and serine proteases, mainly chymotrypsin. The effect of several metal ions on the activity of proteases showed that NaCl, CaCl_2_, CoCl_2_ (5 and 10 mM), and MnCl_2_ (5mM) reduced the protease activity. The kinetic parameters of trypsin-like proteases using N-benzoyl-L-arg-p-nitroanilide as substrate indicated that the *K_m_* and *V_max_* values of trypsin in the alimentary canal were 50.5 ± 2.0 µM and 116.06 ± 1.96 nmol min^-1^ mg^-1^ protein, respectively. Inhibition assays showed only small amounts of cysteine proteases were present in the *G. pyloalis* digestive system. The midgut digestive protease system of *G. pyloalis* is as diverse as that of any of the other polyphagous lepidopteran insect species, and the midgut of larvae contains mainly metalloproteases. Moreover, serine proteases and chymotrypsin also play main roles in protein digestion. Characterization of the proteolytic properties of the digestive enzymes of *G. pyloalis* offers an opportunity for developing appropriate and effective pest management strategies via metalloproteases and chymotrypsin inhibitors.

## Introduction

Proteases are very important enzymes in insects, as they hydrolyze the peptide bonds in dietary proteins to liberate the amino acids needed for growth, survival, and reproduction, and because they detoxify protein toxins ingested as a consequence of plant and pathogen feeding (Terra et al. 1996). The lepidopteran larvae need a proteolytic enzyme complex including trypsins, chymotrypsins, elastases, cathepsin-B like proteases, aminopeptidases, and carboxypeptidases for protein digestion, and many serine proteases are dominant in the larval gut (Patankar et al. 2001; Srinivasan et al. 2006; Chougule et al. 2008; Tabatabaei et al. 2011). Since there is significant variation among the biochemical properties of insect digestive proteases, their characterization is necessary for designing a safe control strategy that utilizes plant-proteinaceous inhibitors (Wilhite et al. 2000). Disruption of protein digestion by these inhibitors has the potential to control insect pests by reducing amino acid availability or by increasing the pests' susceptibility to toxic proteins. Protease inhibitors have been used for a long time for making transgenic plants resistant to pests. Their use in plant protection is notable based on several reports on proteinaceous proteinase inhibitors that represent an alternative approach to pest control. Also, an important factor in activation of *Bacillus thuringiensis* toxin is the presence of specific serine proteases in the midgut microenvironment. There are many potential proteolytic cleavage sites within the activated *Bt* toxin (Kirouac et al. 2006) that further their cleavage by proteases and could either enhance or inhibit *Bt* toxin activity.

The lesser mulberry pyralid, *Glyphodes pyloalis* Walker (Lepidoptera: Pyralidae), is a serious pest of mulberry trees in northern provinces of Iran, especially Guilan province. This pest is a specialist insect on mulberry, *Morus* spp. L. (Rosales: Moraceae), and is widely distributed throughout Asia and the northern provinces of Iran. Because of the importance of mulberry trees in areas such as soil protection, decorative arrangement, renewed resource of valuable timber, and especially the importance of mulberry leaves for the silk industry, protection of these trees against pests should be considered (Madyarov 2008). Since *G. pyloalis* feeds solely on mulberry leaves, it causes serious problems for the silk industry in the north of Iran. In order to combat *G. pyloalis*, use of chemical insecticides should be restricted because they leave residues and insects can develop resistance to them. The residues interfere with the use of the leaves as a diet for silk worms, so insecticide application should be at minimum in sericulture. Alternative methods for pest control that are less hazardous to the environment are highly needed. One area that can be targeted in the development of new insecticidal technologies is the physiology and digestive biochemistry of the insect midgut. The gut physiology of *G. pyloalis* is an important subject for study and the design of new approaches for its control, such as developing transgenic plants that express proteinase inhibitors. Plants have protease inhibitors that mediate plant defenses against herbivores by inhibiting their midgut proteases, thus causing a reduction in the availability of amino acids necessary for their growth, survival and reproduction (Volpicella et al. 2003). Therefore, in this study biochemical properties of digestive proteases of *G. pyloalis* were characterized, and the effects of various inhibitors on enzyme activities were studied, with the aim of identification and application of new pest management technologies. There is currently no information available on the midgut proteases of *G. pyloalis*, and knowledge of its digestive physiology could provide new opportunities for sustainable pest management.

## Materials and Methods

### Chemicals

Azocasein, BAPNA (N-benzoyl-L-arg-p-nitroanilide), BTEE (N-benzoyl-L-tyrosine ethyl ester), TLCK (N-p-tosyl-L-lys chloromethyl ketone), TPCK (N-tosyl-L-phe chloromethyl ketone), PMSF (phenyl methane sulfonyl floride) and iodoacetate were purchased from Sigma-Aldrich (www.sigmaaldrich.com). Trichloroacetic acid and EDTA (ethylene diamine tetraacetic acid) and other chemicals were obtained from Merck Company (www.germany.merck.de).

### Insects

Insects were collected from mulberry trees in Rasht, Guilan province of Iran. They were reared on fresh mulberry leaves at laboratory conditions at 25 ±1° C and 70 ±10% RH, with a photoperiod of 16:8 L:D in transparent clear plastic containers (18 × 15 × 7 cm). Larvae of the same age from the third, fourth, and fifth instars were randomly selected for dissection.

### Sample preparation

Larvae were immobilized on ice and dissected under a stereo-microscope in ice-cold distilled water. The salivary glands, whole gut, foregut, midgut, and hindgut were separately removed, placed in distilled water, and cleaned of adhering unwanted tissues, including the Malphigian tubules and gut contents. The samples were transferred to the freezer (-20° C), stored for 2 weeks, and then homogenized in cold, double-distilled water using a handheld glass grinder on ice (1 gut/10 µl). The homogenates were centrifuged at 20000 ×g at 4° C for 10 min. The resulting supernatants were transferred to new tubes and frozen at 20° C for further use.

### Protease assays

The total protease activities were determined by azocasein digestion method. The assay mixture contained 50 µl of 1% azocasein solution in 25 mM acetate-phosphate-glycine buffer (pH 10.0), 15 µl of buffer, and 10 µl of midgut preparation (protein concentration: 2.4–2.9 mg/mL). After incubation for 90 min at room temperature, the reaction was stopped by addition of 50 µl of trichloroacetic acid. Precipitation was achieved by cooling at 4° C for 30 min, and the reaction mixture was centrifuged at 20000 ×g for 10 min. An equal volume of 1 N NaOH was then added to the supernatant. The absorbance was measured at 440 nm and then converted to units of protease activity by the following equation: (absorbance/extinction coefficient) × 10^3^ = micromoles of dye. The activity of protease was expressed as µmol dye/min/mg protein.

Tryptic activity was measured using chromogenic substrate BApNA. A reaction mixture consisted of 10 µl enzyme, 85 µl of acetatephosphate-glycine buffer (pH 10.0), and 5 µ of the substrate. The absorbance of the samples were then measured at 410 nm by continuously monitoring the change in absorbance p-nitroaniline release for 10 min at 25° C with a microplate reader (Stat Fax® 3200, Awareness Technology, www.awaretech.com).

Chymotryptic activity was assayed according to Hummel (1959) using 1 mM BTEE as substrate dissolved in 50% methanol (v/v), and in 0.08 M Tris-HCl (pH 7.8) containing 0.1 M CaCl2 at room temperature. The increase in absorbance at 256 nm due to the hydrolysis of the substrate was recorded by monitoring the absorption at the wavelength. The aforementioned assays were carried out in triplicate, appropriate blanks were run for all assays.

### Effect of temperature and pH on enzyme activity

The total protease activity was determined by incubating the reaction mixture at different temperatures ranging from 20 to 80° C. The optimum pHs of total protease and trypsin were also determined using a mixed buffer containing citrate (buffer capacity: 2.2–6.5), phosphate (buffer capacity: 5.8–8.0), Tris-HCl (buffer capacity: 7.0–9.0), and glycine (buffer capacities: 2.2–3.6 and 8.8–10.6) (50 mM of each) adjusted on various pHs. Assays were carried out as described above.

### Effects of inhibitors and metal ions on protease activity

The effects of various protease inhibitors such as serine protease inhibitor, 5 mM PMSF; cysteine protease inhibitor, 5 mM idoacetate and 5 mM iodoacetic acid; trypsin inhibitor, 1 mM TLCK; chymotrypsin inhibitor, 1 mM TPCK; and metalloprotease inhibitor, 2 mM EDTA, on proteolytic activities of whole gut enzyme extract were investigated. Furthermore, the effects of chloride salts of various metal ions in 5 and 10 mM on the enzyme activity were also examined. After 30 min of pre-incubation of inhibitors and metal ions with enzymes at room temperature, substrate was added, and residual protease activity was measured by the standard assay method.

### Kinetic parameters of trypsin

The Michaelis—Menten constant *(K_m_)* and the maximum reaction velocities *(V_max_)* of trypsin were determined by Lineweaver—Burk plots. The measurements were carried out at pH 11.0, measuring initial rates of reaction with increasing substrate concentrations. BAPNA was used as substrate at a final concentration range of 0.0156–1 mM. The experiments were performed in triplicate.

### Protein concentration

Protein concentration was measured by the method of Bradford (1976), using bovine serum albumin as the standard.

**Figure 1.**
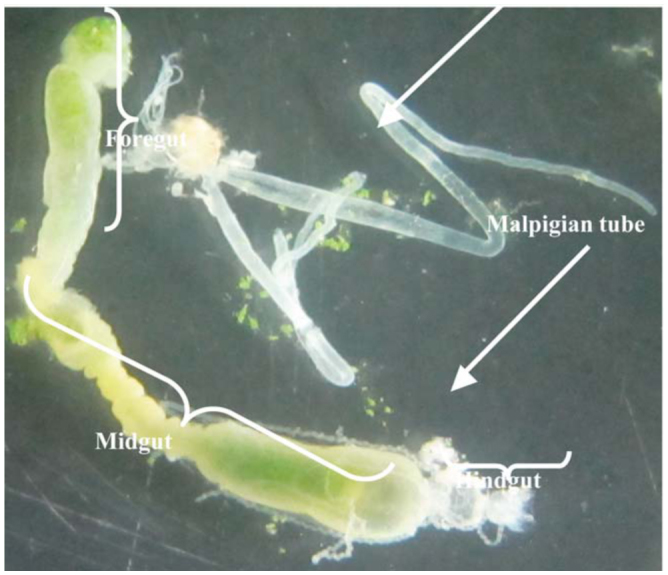
Different parts of the digestive system and salivary glands in the larvae of *Glyphodes pyloalis.* High quality figures are available online.

**Figure 2.**
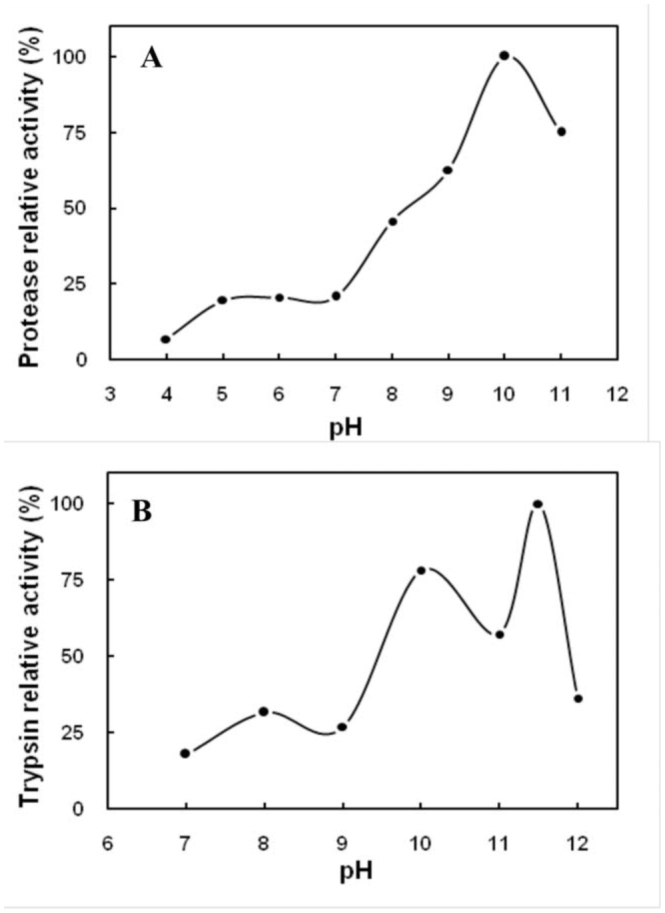
The effect of pH on total protease (A) and trypsin (B) activities extracted from digestive system of larvae *Glyphodes* *pyloalis.* Activities were determined in the mixed buffers adjusted to different values of pH at room temperature. The relative activities were based on the ratio of the activity obtained at a certain pH to the maximum activity obtained at that range and expressed as a percentage. High quality figures are available online.

### Statistical analysis

Data were compared by one-way analysis of variance (ANOVA), followed by Tukey's test when significant differences were found at *p*= 0.05 using SAS program (www.sas.com).

## Results

### Protease activities

Total protease activities in the salivary glands, foregut, midgut, and hindgut were significantly different, with the highest activity in the foregut and midgut (Table 1). The protease specific activities in the digestive system of fifth instar larvae were not significantly different from those of fourth instar larvae. The presence of trypsin- and chymotrypsin-like proteases was shown in larval digestive extracts and salivary glands, by using BAPNA and BTEE as specific substrates. Trypsin activities were significantly higher in the midgut and increased as the larvae aged. Chymotrypsin activity was significantly higher in the salivary gland and foregut than in the midgut or hindgut and was highest in the fifth instar.

**Figure 3.**
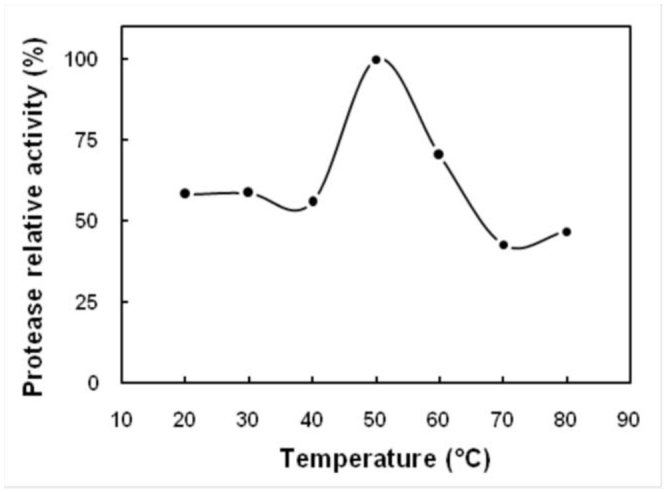
The effect of temperature on total protease activity extracted from digestive system of fifth instar larvae of *Glyphodes pyloalis.* Activities were determined in 20 mM Tris-HCl buffer, pH 7.4, at different temperatures. The relative activities were based on the ratio of the activity obtained at a temperature to the maximum activity obtained at that range and expressed as a percentage. High quality figures are available online.

### Effect of pH and temperature on protease activites

The influence of pH and temperature on the activity of total protease of whole guts (when azocasein was used as a substrate) are plotted in Figures 2 and 3. As is shown, the optimal pH was 10.0, and the optimal temperature was 50° C. The substrate was hydrolyzed over broad ranges of temperatures (20–70° C) and alkaline pHs (pH 8.0–11.5). In the case of trypsin using BAPNA as a substrate, two pH optima were observed at 10.0 and 11.5 (Figure 2). The enzyme activity in the pH range of 7.0–9.0 was relatively low, and maximum activities were observed at a pH range of 9.0– 12.0.

**Figure 4.**
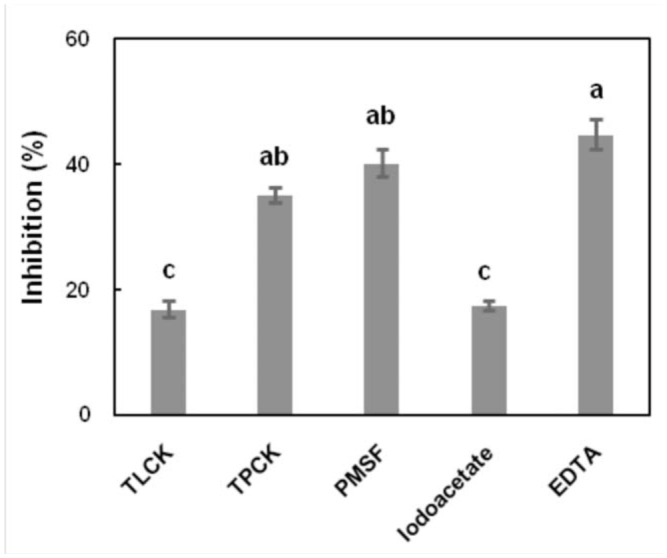
Inhibition percentages of azocasein hydrolysis in presence of various specific protease inhibitors in fifth instar larvae *Glyphodes pyloalis.* High quality figures are available online.

### Effect of metal ions on protease activity

The effects of some metal ions on the enzyme activity are shown in Table 2. The results showed that most metals had little effect on enzyme activity, but NaCl, CaCl_2_, CoCl_2_ (at 5 and 10 mM), and MnCl_2_ (at 5mM) significantly reduced protease activity.

### Kinetic parameters

Kinetic analysis of trypsin-like activity at pH 10.0 gave linear reciprocal Michaelis-Menten (Lineweaver-Burk) plots, enabling estimation of values for *K_m_* and *V_max_.* Kinetic parameters of trypsin in the alimentary canal of fifth instar larvae were measured using BAPNA as substrate. The *K_m_* and *V_max_* values of trypsin in the alimentary canal were 50.5 ± 2.0 µM and 116.06 ± 1.96 nmol min^-1^ mg^-1^ protein, respectively.

### Enzyme inhibition assays

Larval midgut proteases were further characterized using protease specific inhibitors. Calculating of inhibition percentage of azocasein hydrolysis in the presence of various inhibitors offers information about the relative contribution of the inhibited class of protease to total gut protease activity. As shown in Figure 4, EDTA, TPCK and PMSF had the most inhibitory effect on the proteases (∼40% inhibition).

## Discussion

Insects have all the mechanistic classes of proteolytic enzymes that exist in vertebrates (Reeck et al. 1999). These enzymes play important roles in insects' growth, reproduction, development, and health (Terra et al. 1996). The results obtained in this research showed the presence of digestive proteases in the third, fourth, and fifth instar larvae of *G. pyloalis.* The activity of digestive total proteases, trypsin-like and chymotrypsin-like, assayed in the active feeding instar of larvae, expressed as µmol min^-1^ mg^-1^ protein and µmol min^-1^ mL^-1^ per tissue, showed a noticeable increase in tryptic activity between the third and fifth instar. However, total proteases and chymotrypsin activity in fifth instar larvae were higher than in third and fourth instar larvae (Table 1). Previous studies on insect proteases have shown that the main catalytic types of proteases present in insects are serine, aspartate, metalloprotease, and cysteine proteases (Sajid et al. 2002), and in the case of lepidopteran species, trypsin, chymotrypsin, and elastase-like proteinases, aminopeptidases, and carboxy-peptidases comprise the gut proteolytic profiles (Terra and Ferreira 1994). Characterization of the proteolytic activity in digestive system of *G. pyloalis* larvae showed the presence of proteases of different mechanistic classes for protein digestion, with metalloproteases as the dominant form (of those sensitive to the metalloprotease inhibitor tested), and the preferred mode in lepidopteran insects for food digestion is by serine proteases (Patankar et al. 2001; Josephrajkumar et al. 2006; Chougule et al. 2008).

It is not surprising that in spite of similarity in the major classes of proteases present in most lepidopteran insect guts, there is still the possibility of huge variation in their individual gut proteolytic activities. Our results showed that chymotrypsin has a more important role than trypsin in protein digestion in *G. pyloalis *midgut extract, and, in many insect taxa such as Hymenoptera, Coleoptera, Orthoptera, Diptera, and Lepidoptera, the same distributions of these two enzymes have been reported (Gooding et al. 1969; Applebam 1985). The differences in the main catalytic types of proteases between insect species may refer to their phylogenetic relationships or their responses to different diets. Use of inhibitors showed the importance of trypsin in the midgut of the cerambycid *Osphranteria coerulescens* (Sharifi et al. 2012). Biochemical characterization of midgut digestive proteases from the cabbage moth, *Mamestra brassicae*, showed that serine proteinases were predominate, as is typical for lepidopteran larvae, with chymotrypsin-like and trypsin like activities being responsible for approximately 62% and 19% respectively of the total proteolytic activity towards a non-specific protein substrate. Only small amounts of elastase-like activities could be detected in the midgut of *M. brassicae* (Chougule et al. 2008). In contrast, Budatha et al. (2008), using a combination of proteinous substrates and protease specific inhibitors, photometric assays, and activity blots showed 3 trypsin-like serine proteases and one elastase-like serine protease in the midgut of the noctuid moth, *Achaea janata*, but no chymotrypsin-like activity was detected in the digestive system.

Like most enzymatic reactions, the rate of azo dye release from azocasein by *G. pyloalis* proteases increases as the temperature is raised. In the case of these enzymes, as in other enzymes, activities are adversely affected by high temperatures (above 50° C). As shown in Figure 3, digestive system extracts of *G. pyloalis* had azocaseinolytic activity within a broad range of temperatures (20–70 °C), with an optimum temperature of 50° C, while optimum temperatures for protease activity from some Lepidoptera were obtained at temperatures of 30–40 °C (Josephrajkumar et al. 2006; Budatha et al. 2008).

Protease activity was observed at optimal alkaline pH (pH 9.0–12.0). In acidic conditions (pH 4.0 and 5.0), only negligible activity was observed. In highly alkaline conditions (pH 12.0), approximately 78% of maximum activity was recorded. Alkaline pH values for activity are due to intrinsic alkaline pH of the insect digestive system, and have been reported for many lepidopteran insects ( Purcell et al. 1992; Ferry et al. 2005; Budatha et al. 2008; Chougule et al. 2008). Tabatabaei et al. (2011) showed that proteolytic activity of the carob moth, *Ectomyelois ceratoniae*, with hemoglobin as protein substrate occurred over a broad alkaline pH range (pH 8.0–11.0), with maximum activity at pH 10. Furthermore, the pH optima obtained for trypsin activity using its specific substrate, BAPNA, was at the alkaline end of the spectrum (pH 10.0 and 11.5), which is similar to many lepidopteran insects (Purcell et al. 1992; Ferry et al. 2005; George et al. 2008). In *M. brassicae*, trypsin activity, as assessed using *N_a_*-benzoyl-DL-arginine *p*-nitroanilide, exhibited a peak at pH 7.5 and activity from pH 9.0–11.0. Chymotrypsin activity, assessed using *N_a_*-succinyl-alanylprolyl-phenylalanine *p*-nitroanilide, also peaked at pH 7.5, with a second activity peak at pH 11.0 (Chougule et al. 2008). In our research, trypsin of *G. pyloalis* exhibited a peak at pH 10.0 and optimum activity at pH 11.5, suggesting the presence of different isoforms of this enzyme. Midgut proteinases of *E. ceratoniae* hydrolyzed the synthetic substrates of trypsin, chymotrypsin, and elastase at pH 8.0– 11.0 (Tabatabaei et al. 2011). The pH activity profile of the trypsin-like in *G. pyloalis* is different from that of other lepidopteran larvae, which show a pH optimum below 11.0 (Ahmed et al. 1980; Johnston et al. 1991, 1995; Purcell et al. 1992; Gatehouse et al. 1999).

The effects of metal ions on protease activity showed that chloride salts of Na, Ca, and Co at 5 and 10 mM and Mn at 5 mM reduced azocaseinolytic activity, whereas the majority of metals had no effect on enzyme activity. However, metals do have an effect on larvae of another lepidopteran, namely *Conogethes punctiferalis* (Josephrajkumar et al. 2006).

The *K_m_* and *V_max_* values of trypsin in the alimentary canal of *G. pyloalis* using BAPNA as substrate were 50.5 ±2 µM and 116.06 µ1.96 nmol min^-1^ mg^-1^ protein, respectively. *K_m_* and *V_max_* values for *M. brassicae* trypsin were determined using Z-Arg-7-amido-4-methylcoumarin hydrochloride, a fluorescent substrate, as 69 µM and 383,000 nmol min^-1^ ^-1^ protein, respectively (Chougule et al. 2008). The high *V_max_* value for *M. brassicae *trypsin is justified by using high sensitive substrate (measured with fluorometry technique) and the major activities of serine proteinases in midgut of this pest, whereas, midgut of *G. pyloalis* contains metalloproteases as the dominant proteases. Also, trypsin *K_m_*, and *V_max_* values in the alimentary canal of *O. coerulescens* were reported as 690 µM and 560 nmol min^-1^ mg^-1^ protein, respectively, when BAPNA was used as substrate (Sharifi et al. 2012). Trypsin from the midgut of *Helicoverpa armigera* had a *K_m_*, of 2300 µM and *V_max_* of 430 nmol min^-1^ mg^-1^ protein with BAPNA as substrate (Ozgur et al. 2009). The *K_m_*, value of trypsin in midgut of *Eurygaster integriceps *determined using BAPNA as substrate was 600 µM (Hosseininaveh et al. 2009). The *K_m_* value reported in the midgut of other insects was 80–930 µM (Tsybina et al., 2005).

The biochemical analysis of *G. pyloalis* larval digestive enzymes using specific peptide substrates and inhibitors showed that metalloproteases and serine proteinases were predominate in digestive systems of fifth instar larvae. The presence of chymotrypsin and trypsin-like enzymes in *G. pyloalis* larval gut was clearly demonstrated by the hydrolysis of chymotrypsin and trypsin specific substrate (BTEE and BAPNA) and inhibition of proteolytic activity by the chymotrypsin and trypsinspecific chemical inhibitor TPCK and TLCK, respectively. Slight inhibition of protease activity (17.4%) by iodoacetamide occurred, suggesting that cysteine proteinases were slightly responsible for protein digestion in the midgut of *G. pyloalis.* These data suggest that the midgut of larvae contains mainly metalloproteases; moreover, serine proteases and chymotrypsin also have important roles in protein digestion. TLCK has a considerably lower inhibitory effect on hydrolyzing azocasein compared to TPCK (about 18% less), suggesting that chymotrypsin has a more important role than trypsin in protein digestion in the larval midgut. The inhibitory effect of idoacetate was also very low. Azocaseinase gut activity in the Indian meal moth, *Plodia interpunctella*, at pH 9.5 was inhibited by serine proteinase inhibitors SBTI (soybean trypsin inhibitor) and TLCK, which are specific to trypsin-like enzymes, at 96% and 89%, respectively. In this insect, protease activity was inhibited by iodoacetamide, a cysteine protease inhibitor, TPCK, a chymotrypsin inhibitor, and EDTA, a metalloprotease inhibitor, at 47%, 8%, and 28%, respectively (Amorim et al. 2008).

In conclusion, the midgut digestive protease system of *G. pyloalis* is as diverse as that of any of the other polyphagous lepidopteran insect species, with metalloproteases and chymotrypsin used for food digestion. Plant proteinase inhibitors may inhibit different classes of insect peptidases, including serine, cysteine and aspartate proteinases, and metallocarboxypeptidases, although most of plant proteinase inhibitors that are known and characterized so far interact with trypsin and chymotrypsin proteinases (Terra and Ferreira 1994; Bode and Huber 2000). The knowledge gained about protease enzyme classes can be used to evaluate the response of *G. pyloalis* to proteinaceous inhibitors using metalloprotease and chymotrypsin proteinase classes as digestive enzyme targets. Characterization of the proteolytic properties of the digestive enzymes of *G. pyloalis* offers an opportunity for developing appropriate and effective pest management strategies through protease inhibitors.

**Table 1.**
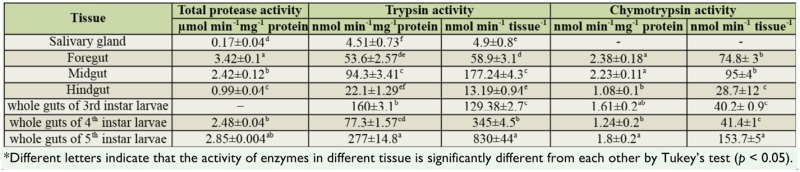
Mean activity level (± SE) of total protease, trypsin, and chymotrypsin in different parts of digestive system and salivary glands of fifth instar larvae and whole gut of fourth and fifth instar larvae.

**Table 2.**
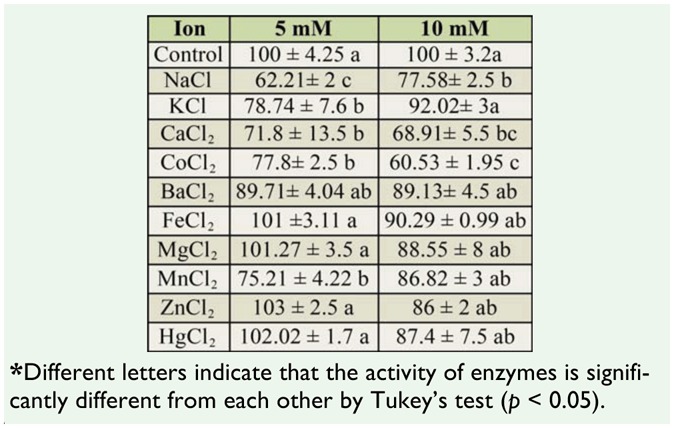
The effect of various metal ions (5 and 10 mM) on total protease activity (mean ± SE) extracted from midguts of fifth instar larvae *Glyphodes pyloalis.*

## Abbreviations

**BAPNA**,: N-benzoyl-L-arg-p-nitroanilide
**BTEE**,: N-benzoyl-L-tyrosine ethyl ester
**EDTA**,: ethylene diamine tetraacetic acid
*Km*,: Michaelis-Menten constant
**PMSF**,: phenyl methane sulfonyl floride
**TLCK**,: N-p-tosyl-L-lys chloromethyl ketone
**TPCK**,: N-tosyl-L-phe chloromethyl ketone
*V_max_*,: maximum reaction velocity
